# Tubulin posttranslational modifications as regulators of microtubule-based functions in cells

**DOI:** 10.1186/2046-2530-1-S1-P17

**Published:** 2012-11-16

**Authors:** M Bosch-Grau, C Rochas, A Meunier, N Spassky, C Janke

**Affiliations:** 1 Institute Curie, France; 2 Biology Institute of the Ecole Normale Superieure, France

## 

Microtubules (MT) form a dynamic intracellular network that plays essential roles in cellular function and development. They are also the structural components of cilia and flagella. MTs maintain cilia structure and dynamics by the specific interaction of a large set of MT-associated proteins, including molecular motors and MAPs. How the interactions between MTs and their multiple associated proteins are spatio-temporally is not known. An increasing body of evidence supports that tubulin posttranslational-modifications (PTMs) have a key role in regulating the recruitment of protein complexes in the MT because they allow for a rapid, reversible and locally restricted generation of MT diversity. In particular, two PTMs, glutamylation and glycylation are strongly enriched in the cilium. Glycylation is restricted to axonemal MTs, suggesting a highly specialised function, whereas glutamylation is also present in centrioles and basal bodies. Our hypothesis is that differentially glutamylated/glycylated MTs can distinguish MT subpopulations in the cilium allowing for the recruitment of specific proteins. Here we address the role of glutamylation and glycylation in cilia, by modulating the extent of each modification using the ependymal cell system as a model. We study the role of both modifications in cilia physiology, including cilia assembly, maintenance and beating. Our data demonstrate that glycylation is essential for cilia maintenance, whereas glutamylation is required for cilia beating. Taken together, tubulin PTMs are likely to play an important role for cilia and could possibly been linked to human disorders known to be associated with defects in MT-based traffic, such as ciliary dysfunctions (ciliopathies).

